# Assessing the Chronic Effects of Dietary Aluminum on Fitness Traits, Acetylcholinesterase Activity and Locomotion in *Lymantria dispar* L. Larvae

**DOI:** 10.3390/insects16111146

**Published:** 2025-11-09

**Authors:** Milena Vlahović, Dragana Matić, Dajana Todorović, Branka Petković, Larisa Ilijin, Marija Mrdaković, Vesna Perić-Mataruga

**Affiliations:** 1 Department of Insect Physiology and Biochemistry, Institute for Biological Research “Siniša Stanković”, National Institute of the Republic of Serbia, University of Belgrade, Despot Stefan Blvd 142, 11108 Belgrade, Serbia; dragana.matic@ibiss.bg.ac.rs (D.M.); dajana@ibiss.bg.ac.rs (D.T.); lararid@ibiss.bg.ac.rs (L.I.); mm1507@ibiss.bg.ac.rs (M.M.); vesper@ibiss.bg.ac.rs (V.P.-M.); 2 Department of Neurophysiology, Institute for Biological Research “Siniša Stanković”, National Institute of the Republic of Serbia, University of Belgrade, Despot Stefan Blvd 142, 11108 Belgrade, Serbia; janac@ibiss.bg.ac.rs

**Keywords:** aluminum, *Lymantria dispar* larvae, acetylcholinesterase, fitness traits, locomotion

## Abstract

*Lymantria dispar* larvae were used as a model to study the effects of sublethal chronic dietary aluminum exposure at concentrations of 50, 250, 500, and 1000 μg Al/g dry food (T1, T2, T3, and T4, respectively) on fitness traits, acetylcholinesterase (AChE) activity, and locomotion. At the lowest concentration, the greatest reduction in mass and AChE activity, as well as an increase in travel distance and time in movement, was observed relative to the control group. Strong positive correlations between larval mass and time in movement were observed in T1. AChE activity correlated positively with larval mass and relative growth rate (RGR) and negatively with locomotion parameters. An increase in relative growth rate led to a decrease in locomotion parameters (250, 500, and 1000 μg Al/g dry food). As the amount of metal increased, there was a trend towards a decrease in locomotion, and the value was observed to approach the control value. This study suggests that aluminum has an influence on the fitness characteristics, AChE activity, and locomotion of *L. dispar* larvae. The results obtained allow us to better understand the toxicity of aluminum and, at the same time, underline the need for further studies involving insects.

## Simple Summary

Aluminum is one of the most abundant elements in the Earth’s crust. However, many organisms are excessively burdened by this element, as air, water, food, medicines, cosmetics, and pesticides all contain aluminum. Numerous studies have demonstrated the toxicity of aluminum, especially to humans and other mammals, while experiments with insects are very limited and rare. To the best of our knowledge, there are no data describing the effect of aluminum on *Lymantria dispar* (Lepidoptera: Erebidae), one of the most widespread and harmful phytophagous species. In this study, the chronic effects of four dietary aluminum concentrations on larvae from hatching to the fifth larval instar were investigated. We monitored changes in fitness traits (mass, relative growth rate, and days of development), acetylcholinesterase (AChE) activity, and locomotion parameters. Our results show that chronic dietary aluminum exposure affects fitness traits and behavior, AChE activity, and locomotion. There is a negative correlation between enzyme activity and locomotion. These results demonstrate that aluminum affects the tested traits of this well-adapted insect species; further studies are needed to determine potential biomarkers of aluminum exposure in insects.

## 1. Introduction

In recent years, the potential damage caused by aluminum has become the subject of an increasing number of analyses. Aluminum (Al) is the most abundant metal in the Earth’s crust [1]. Due to anthropological influences, this metal is very widespread in nature, so various types of studies are needed to explain its effects on living organisms. The presence of aluminum in water, food, medicines, and vaccines leads to constant exposure for living organisms. For example, aluminum sulfate is used to remove organic contaminants from drinking water, and this is only one source of daily exposure to this element [2,3,4,5]. Due to its toxicity, posing a real threat but one that is not immediately detectable and whose mechanisms are not understood, some scientists refer to it as a “silent killer” [6]. Therefore, it is necessary to understand its influence on various organisms, the potential damage it can inflict, methods of detoxification, and the initial visible changes in organisms in the presence of aluminum.

Aluminum is associated with Alzheimer’s disease [7,8,9]. The cholinergic system is very sensitive to metal exposure; this is especially true for AChE [10]. Most studies in this vein focus on the influence of aluminum on the activity of cholinesterases. These enzymes represent a family that catalyzes the hydrolysis of the neurotransmitter acetylcholine (ACh). During this reaction, ACh is broken down into choline and acetic acid. Acetylcholinesterase (AChE; E.C. 3.1.1.7) is found in nerves, muscles, and various types of fibers and tissues [11]. Higher AChE activity has been found in motor neurons. The degradation of AChE can precipitate overbinding of ACh and overstimulation of postsynaptic neurons, resulting in memory loss, hyperkinesia, and excessive autonomic nervous system activity [12,13,14]. AChE is sensitive to the presence of heavy metals, so it can be used in screening for environmental pollutants. So far, research on the influence of metals and AChE has mainly focused on the human population. We believe that studying the impact of aluminum on phytophagous species is very important because of their position in the food chain and the response of the gypsy moth population itself. Furthermore, it is not yet clear whether the same mechanisms of aluminum-induced neurological disorders occur in mammals and terrestrial insects. The studies to date are quite contradictory. Some authors have demonstrated a decrease in AChE activity [15,16], while other studies have found increased activity of the same enzyme [17,18]. The AChE activity, motility, and color susceptibility of honeybees were found to be dependent on the concentration of aluminum, the duration of the metal’s effect, and the subspecies of insect examined [19].

Diet is the main route of aluminum exposure for the general population [20]. Exposure leads to neurodegeneration, the consequences of which are memory loss and changes in behavior, movement, and learning [21]. The effects of aluminum on the brain and the mechanism of toxicity are still under debate. In rare experiments describing the effect of aluminum on insects, this metal was found to affect the lifespan, locomotor activity, and circadian rhythm of *Drosophila melanogaster* [19]. Data describing the effect of aluminum on fitness traits (mass, relative growth rate, and developmental duration) in animals are scarce. There are a few experiments pertaining to honeybees, mealworms, fruit flies, and bumblebees [19,22,23,24,25]. It has been proven that aluminum has a toxic effect on various developmental stages of *Drosophila melanogaster*. However, the mechanism of toxicity is not yet clear, nor is the reason why some species show resistance to aluminum [25].

There is a dearth of data describing the influence of aluminum on fitness traits in insects, especially in phytophagous species. Thus, the experiment conducted in this study provides a unique set of data describing changes in traits that are easily detected and can serve as early-warning signals in the presence of aluminum. Concentrations of this metal in plants (including numerous edible species) vary between 15 and 3470 μg/g [26], while its concentration in leaves that accumulate the metal exceeds 20,000 μg Al/g [27]. As the aluminum concentration in plants varies, the composition of the leaves has a direct effect on herbivorous insects, which are thus exposed to a high risk. In addition, contaminated plants and insects can be a link through which aluminum enters the terrestrial food chain. Therefore, the knowledge gained from insect models can be applied to more complex organisms, including humans [28]. The gypsy moth is a phytophagous insect group; this is the main reason why the larvae in our study were exposed to aluminum concentrations in a wide range from 50 to 1000 μg/g dry food. Critically, the concentration in cosmetic products intended for daily use is many times higher [29,30]. We could not find any references that consider lethal concentrations for terrestrial insects. Previous studies mostly focused on freshwater species (EC_50_ or LCP_50_). For *Heptagenia* spp., *Ephemera* sp., and *Somatochora* sp., the lethal concentrations are in the range of 2000–20,000 mg/kg [31,32].

The effect of aluminum on arthropods, including insects, has scarcely been studied. As lepidopterans have not yet been used in such studies, the aim of this investigation was to identify the first visible changes in the development of *L. dispar* following dietary aluminum exposure and assess whether this species is suitable as a potential model system for aluminum toxicity. This experiment can be considered a pilot study. We aimed to determine whether the aluminum concentrations used elicited a response in Lepidoptera larvae and whether the key components of this study are feasible and variable. The aim of our research is to determine the chronic effects of four aluminum concentrations on (1) fitness traits, (2) AChE activity, and (3) locomotion in *Lymantria dispar* L. larvae. Furthermore, the question arises as to whether the effects of aluminum are the same in humans and other organisms.

## 2. Materials and Methods

In this study, we analyzed a population of *L. dispar*. Egg masses were collected in late autumn 2022 in the forests of Morović in western Serbia (45° 00′ 26″ N; 19° 12′ 56″ E) and stored at 4 °C until spring. The hairs were removed from the egg hatchlings, and the eggs were mixed and sterilized with 2% sodium hypochlorite solution. The dried eggs were placed in an insect chamber at 23 °C until the larvae hatched. In the 1st instar phase, they were randomly assigned to five experimental groups: a control group (C), which was fed an artificial diet without aluminum, and aluminum-treated groups T1, T2, T3, and T4 (fed an artificial diet with 50, 250, 500, and 1000 μg Al/g dry food, respectively). The larvae were reared individually from the 1st day of the 1st larval instar at a temperature of 23 °C and a relative humidity of 60%, with a 12 h/12 h photoperiod. Larvae were evenly distributed among the experimental groups until they were sacrificed on the 3rd day of the 5th larval stage. The sample sizes of the experimental groups for fitness traits and enzyme activity were C = 19; T1 = 18; T2 = 14; T3 = 17; and T4 = 16, with 9 larvae per treatment for locomotion. The food was replaced every 48 h. We used an artificial diet for insect rearing. Wheat bran, dry yeast, agar, and vitamin complex are the major components of the diet that the insects were fed [33]. The aluminum concentration in the diet was based on the amount of aluminum in the anhydrous salt AlCl_3_ (Fluka, Charlotte, NC, USA). All procedures followed for the use of animals were in accordance with the Ethical Committee for the Use of Laboratory Animals of the Institute for Biological Research “Siniša Stanković”, National Institute of the Republic of Serbia, University of Belgrade.

Once the 3rd day of the 5th instar began, larval mass was measured, and the animals were put on ice and sacrificed. They were decapitated, the midgut was removed through an abdominal incision, and the integument was collected for further analysis. The brains of the larvae were removed from the head capsule on ice using an Olympus KL1500LCD cold-light source (Tokyo, Japan). Each homogenate was prepared from 7–10 brains (pooled homogenate). The brain tissue was diluted with 0.9% NaCl (1:9/*w*:*V*). Homogenization was performed in 0.9% NaCl, employing 3 × 10 s/5000 rpm with 15 s breaks. The homogenates were centrifuged for 10 min/10,000× *g*/4 °C (Eppendorf 5417R centrifuge, Hamburg, Germany) and stored at −80 °C. The homogenates were stored for seven days before experiment. The supernatants were used for analysis.

### 2.1. Fitness Traits

Larval mass was measured on the 1st and 3rd day of the fifth larval instar period.

The relative growth rate was measured from the point of molting into the 5th larval instar (1st day) to the 3rd day of the 5th larval instar. The relative growth rate was calculated as follows [34]:

$$Relative\;growth\;rate=\frac{({mass}_{3}-{mass}_{1})\times 100}{days\times {mass}_{1}}$$

mass_1_ and mass_3_—larval mass at the 1st and 3rd day of the 5th instar, respectively, days—number of days between measuring (2 days).

Development time (days) represents the time from hatching until sacrifice on the 3rd day of the 5th instar.

### 2.2. Enzyme Detection

AChE activity was determined with acetylcholine iodide (0.25 M) [35], which served as the substrate used for the chemical reaction. The enzyme produces thiocholine, which reacts with 5,5′-dithiobis-2-nitrobenzoic acid (DTNB) to form a colored product, the amount of which is proportional to the enzyme activity. A 50 mM sodium phosphate buffer (pH 7.9) was used in the reaction. The reaction mixture contained DTNB dissolved in buffer, acetylcholine iodide dissolved in water, and 15 or 30 μL of homogenate, depending on the amount of protein. The enzyme kinetics were measured at 406 nm/25 °C for 3.5 min with a lag period of 60 s using a Shimadzu UV-1800 spectrophotometer (Kyoto, Japan). In the enzyme assay, two blanks and three replicates were analyzed for each group. AChE activity was expressed as μmol of substrate/min/g tissue [36].

### 2.3. Locomotion Monitoring

Behavioral testing was conducted according to established protocols in an experimental arena large enough to avoid excessive clinging to the wall or restricting the movement of the insect [37]. The *L. dispar* (5th instar) larvae, which were employed for the study of locomotion, were allowed to move freely in Petri dishes (diameter: 85 mm) for 5 min, with one larva per dish. The locomotion of three individuals was recorded simultaneously using a LifeCam VX-6000 (Microsoft, Redmond, WA, USA) placed approximately 30 cm above the Petri dishes in the control room between 9 and 11 am under the same rearing conditions. Each larva was tested only once, and three common parameters of locomotion, namely, travel distance (m), time in movement (s), and average speed while in motion (m/s), were analyzed using Any-maze video tracking software (v.7.2, Stoelting Co., Wood Dale, IL, USA).

### 2.4. Statistical Analysis

The statistical analysis was carried out using the GraphPad Prism 9 program. The mean value and the standard error of the mean (SEM) were calculated for each characteristic analyzed. The Kolmogorov–Smirnov test was used to test normality for all fitness traits, enzyme activity, and locomotion parameters. The homogeneity of variance was tested using the Brown–Forsythe test. Subsequently, a parametric one-way ANOVA, followed by Tukey’s test, was used to assess larval mass, development time, and AChE activity, while the relative growth rate was analyzed using a Welch ANOVA test followed by Dunnett’s test (unequal variances). The Spearman correlation coefficient was calculated using Excel + Analyse-it. A Kruskal–Wallis ANOVA, followed by post hoc Mann–Whitney U-test, was used to analyze differences in locomotion parameters.

## 3. Results

**Figure 1:**
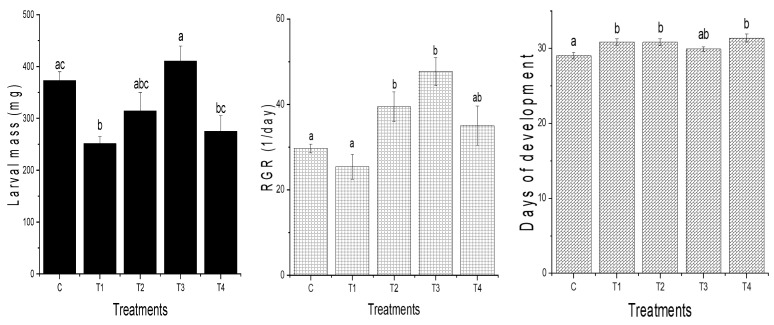
Fitness traits (mass, relative growth rate, and development time) of *L. dispar* larvae exposed to aluminum at concentrations of 50, 250, 500, and 1000 μg Al/g dry food (T1, T2, T3, and T4, respectively) and the control group (C). Each bar represents the mean value ± SEM. Different lowercase letters indicate significant differences (*p* < 0.05) between groups.

**Figure 2:**
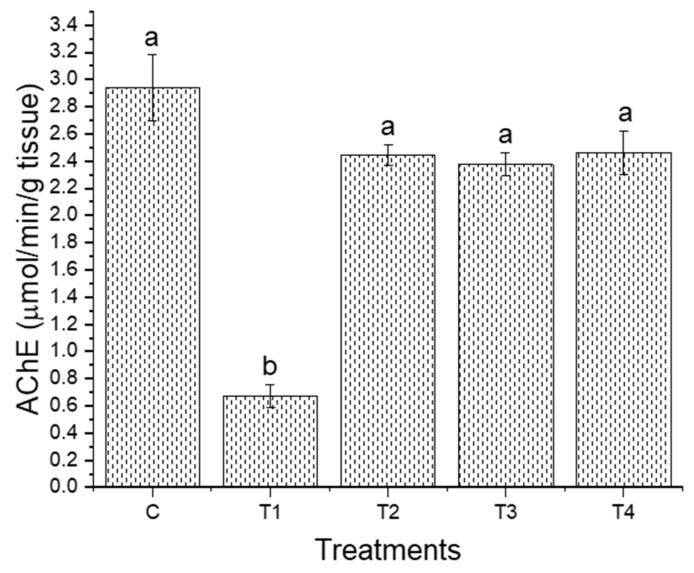
AChE activity in the brains of *L. dispar* larvae exposed to aluminum at concentrations of 50, 250, 500, and 1000 μg Al/g dry food (T1, T2, T3, and T4, respectively) and the control group (C). Each bar represents the mean value ± SEM. Different lowercase letters indicate significant differences (*p* < 0.05) between groups.

Chronic exposure to dietary aluminum exerted different effects on the analyzed fitness characteristics of the *L. dispar* larvae depending on the concentration used (Figure 1). Larval mass was significantly decreased for T1 relative to the control treatment. A significant decrease in the same trait was also detected for T1 relative to T3 and for T4 in comparison to T3. The relative growth rate was significantly increased in groups T2 and T3 relative to the control group and T1, as was the development time in T1, T2, and T4 relative to the control group. The modalities for the change in larval mass and relative growth rate were similar: there was an increase in values for these two fitness traits in T2 and T3 in relation to T1, and there was a decrease in T4 relative to T3 (Figure 1). Interestingly, the largest decrease in these two traits, relative to all the groups, was observed at the lowest aluminum concentration (T1). The mean values of larval mass were significantly different between groups T3 and T4, too. Regarding development time, a statistically significant increase was observed in groups T1, T2, and T4 relative to the control group. There were no significant differences between the control group and the mass or development time at 1000 μg Al/g dry food (T4 group). In contrast to the control larvae, a trend towards an increase in the number of days required to reach the third day of the fifth larval stage was observed for all the aluminum-treated larvae, indicating a unique developmental strategy was adopted by all the groups exposed to aluminum. Apart from significant differences in development time between the control and T1, T2, and T4 treatments, there were no other differences between the aluminum treatments. AChE activity was only significantly reduced, relative to the control group, with 50 μg Al/g dry food (T1 group) (Figure 2). The levels of AChE activity in the larvae treated with higher aluminum concentrations (groups T2, T3, and T4) were similar and slightly reduced relative to the control group (this result is statistically non-significant).

**Figure 3:**
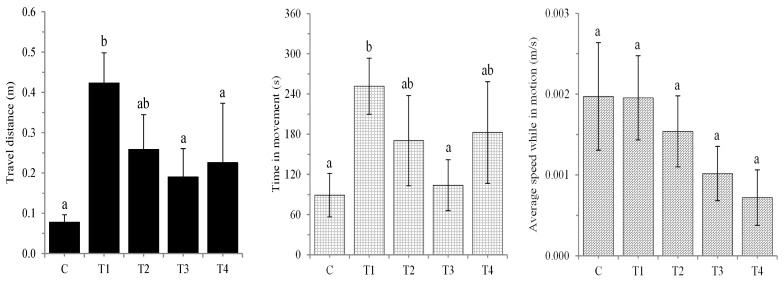
Locomotion (travel distance, time in movement, and average speed while in motion) of *L. dispar* larvae exposed to aluminum at concentrations of 50, 250, 500, and 1000 μg Al/g dry food (T1, T2, T3, and T4, respectively) and the control group (C). Each bar represents the mean ± SEM. Different lowercase letters indicate significant differences (*p* < 0.05) between groups (Mann–Whitney U-test).

The consumption of aluminum-containing food altered the locomotion of the *L. dispar* larvae, showing a characteristic U-shaped dose response (Figure 3). These changes are most clearly reflected in the travel distance parameter and are significant according to the Kruskal–Wallis ANOVA test. The larvae from T1 travelled the longest distance, and this change was statistically significant compared to the control group. The travel distances of the larvae fed 500 and 1000 µg Al/g of dry food (groups T3 and T4, respectively) were statistically lower than those of the larvae fed 50 µg Al/g of dry food. Evidently, the average speed while in motion decreased with an increasing aluminum concentration; according to the Kruskal–Wallis ANOVA test, these differences are close to the level of significance. In this context, a detailed post hoc comparison revealed a statistically significant increase in time in movement for the larvae from T1 relative to the control group and T3.

The sign and strength of the correlation change depended on the treatment. At the highest aluminum concentration, mass was negatively correlated with locomotion parameters (Table 1). Strong positive correlations were found between mass and AChE and time in movement at the lowest aluminum concentration. Importantly, as enzyme activity increased, the time in movement, travel distance, and average speed while in motion decreased for the larvae fed 250, 500, and 1000 μg Al/g dry food. However, the increases in enzyme activity after the T3 and T4 aluminum treatments were positively correlated with larval mass and the relative growth rate. In the same treatments, a decrease in the relative growth rate led to an increase in all larval locomotion parameters (T2, T3, and T4). In addition, at the highest aluminum concentration, the smaller larvae needed more time for full development (a negative correlation).

## 4. Discussion

Our results show that aluminum influences the fitness characteristics, AChE activity, and locomotion of *L. dispar* larvae. Interestingly, for most of the traits tested, the strongest response relative to the control group occurred after chronic treatment with the lowest aluminum concentration (50 μg Al/g of dry food). For these larvae, we observed a statistically significant decrease in mass and AChE activity, as well as an increase in travel distance and time in movement, compared to the control. The relative growth rate increased, relative to the control, at a concentration higher than 50 μg Al/g dry food (this parameter refers to larval growth per unit time) and exhibited almost the same type of change as larval mass. It is very likely that the detoxification mechanisms had not yet been activated and that it is energetically more favorable for an organism to influence the fitness characteristics that are not of crucial importance to it. Decreases in cell viability in vitro and survival and body weight gain in vivo in *Bombyx mori* larvae were reported in [38]. Clearly, the strategy used for defense and survival depends on the metal concentration. It is likely that these processes are based on a trade-off between larval development and mechanisms of detoxification and behavior, enabling optimal energy use under stressful conditions. Numerous biochemical and molecular mechanisms influence the growth and mass of organisms. In wasps, a sharp drop in mass, accompanied by an increase in metabolic rate, has been observed under stress conditions [39]. At the lowest aluminum concentration, the energy required for development, foraging, and locomotion takes priority over mass and growth. Protein synthesis underlies the increase in mass [40], which is evidently more effective at higher sublethal metal concentrations, up to a certain level (1000 μg Al/g dry food in our experiment). In the T2 and T3 treatments, mass and relative growth rate (RGR) increased, but all locomotion parameters reduced, most likely due to energy allocation in order to reach adulthood. The highest concentration of aluminum led to irreversible harmful effects, along with greater variability in plasticity for larval mass and RGR, which probably enables better survival in polluted environments. We can conclude that, depending on the intensity of stress (i.e., the amount of aluminum in their food), insects exhibit various physiological, behavioral, and biochemical adaptations that allow for successful survival. By increasing the number of days in the development process, a larva has more chances to reach sexual maturity as an adult, but, at the same time, the activity of some enzymes (in our case, AChE) remains constant.

The results describing the effect of aluminum on AChE activity are quite contradictory. In contrast to this inhibition at 50 μg Al/g dry food, there are no statistically significant changes at higher concentrations compared to the control. Similar results were obtained regarding honeybees, i.e., a decrease in enzyme activity at low metal concentrations and an increase at higher concentrations [19]. The same alterations were detected by measuring motility, for which we could not detect a linear change depending on the metal concentration. This is not the first time in our research that we have found a stronger response at low metal concentrations compared to that at higher concentrations [41,42,43]. In an earlier study, we found that large quantities of metals are excreted from this organism through the head capsule [44]. It is possible that with the accumulation of aluminum in the head capsules at higher concentrations, a large amount of this metal is excreted from the body, thus reducing its toxic effect. It is important to emphasize that some insect species, mostly freshwater species, can accumulate aluminum in doses that may be toxic to predators. It was found that Plecoptera can contain 4900 mg Al/kg bodyweight, indicating high tolerance and/or the possibility of excretion [45]. In mayflies, 70% of the aluminum load is excreted via the exuviae [46], while 91 times more exuvial aluminum can be detected in comparison to recently molted larvae [47]. This could be one of the reasons for the adaptations of *L. dispar* larvae and the lack of enzyme inhibition and changes in fitness traits at high metal concentrations. Accumulated aluminum was found in the earthworm *Eisenia andrei*, but a positive correlation between the bioaccumulation factor and aluminum concentrations in soils was not detected [48]. In addition, a long-lasting effect and a certain concentration of metals can more easily lead to compensatory reactions that increase an organism’s tolerance within a certain genetic range [49,50]. Experiments conducted in vitro on zebrafish showed that aluminum can lead to an increase in enzyme activity through its direct effect on AChE without directly influencing the signaling pathways [51]. This phenomenon was ascribed to an interaction between the divalent cation and the anionic site of the enzyme [52]. Considering that aluminum causes increased peroxidation of lipids and their disarrangement, it is possible that these changes alter AChE activity during aluminum stress, too [53]. AChE is one of the fastest and most efficient enzymes (hydrolyzing 10^8^ molecules of a substrate per second) [54]. Thus, the slightest change in its activity might have a significant effect on fitness [55]. In our experiment, we clearly demonstrated this, as shown by the inhibition of the enzyme and alterations in larval mass and development time.

Although not statistically significant, a downward trend in enzyme activity relative to the control was observed after the T2, T3, and T4 treatments, along with greater travel distances and time in movement. These data are supported by the negative correlations between these traits. The greater AChE activity at 500 and 1000 μg Al/g of dry food compared to 50 μg Al/g of dry food was accompanied by increases in mass and RGR. It is possible that under high stress, all resources are used for larval survival and transition to the adult stage, although these two parameters (enzyme and mass) are not directly linked. In addition, *Lucilia sericata* is more susceptible to toxicity during adult development as opposed to larval development with respect to exposure to cadmium [56]. Therefore, the harmful effects of these metals and a decrease in adaptive capacity are only observed later in the adults. In addition, aluminum has a cumulative effect [57]. In one study, a type of resistance that develops during aluminum exposure was discovered in flies [24]. Individuals that survived the first week of exposure showed fewer behavioral changes than younger flies. The same authors found that the lifespan and sensitivity of locomotor activity depended on age and sex. With an increase in concentration and prolonged exposure, the metal accumulates in the body up to a certain limit. However, when the amount of metal reaches the “critical point”, the detoxification mechanisms are no longer able to respond appropriately, and the parameters studied (mass, relative growth rate, travel distance, and time in movement) change. In addition, the larvae of the gypsy moth live on more than 500 plant species, some of which are metal hyperaccumulators. It is likely that these insect species are well adapted to the presence of metals, and this could be why we did not find statistically significant differences between the control and the treatments with higher aluminum concentrations in the parameters studied, except for relative growth rate and development time. Furthermore, the process known as hormesis should be considered. It occurs during exposure to low doses of a stressor; under these conditions, the stressor induces a stimulatory effect with respect to an examined parameter and inhibition at higher doses [58]. Hormesis is an adaptive trait and can either be the result of compensatory biological processes necessary for the maintenance of homeostasis or be directly induced [59,60]. In this way, the initial adverse effect of low concentrations of xenobiotics is overcome. This phenomenon was also relevant in our previous research, as mentioned above. Regarding locomotion, a hormetic or U-shaped dose response was observed for travel distance and time in movement, but not for average speed while in motion. This finding is somewhat expected, as hormesis is a complex phenomenon whose manifestation depends on the parameter being measured, its variability, and the magnitude of the response to a given treatment [61]. Therefore, it can also be proposed that aluminum-induced changes in travel distance are directly related to time in movement and do not depend on average speed while in motion. Enzyme insensitivity to aluminum in the gypsy moth suggests resistance to certain xenobiotics to maintain stable homeostasis [62]. In addition, the activity of this enzyme is also governed by the pollution of the environment from which the egg masses originate [63]. This fact is not recognized by many researchers, and it could explain the different responses of AChE during exposure to stressors. It is possible that there are (pre)adaptations to the stressor that cause the lack of an enzyme response at lower concentrations of the xenobiotic tested. In addition, some metals (silver, lead, zinc, and cadmium) led to only 10% inhibition of AChE in *Periophthalmodon schlosseri* [64].

Aluminum binds to chromatin and changes gene activity, reduces transcription and protein synthesis, and blocks normal organ development by reducing cell divisions [65]. All of these factors may be direct or indirect causes of the changes in mass and growth rate that require new regulatory and anabolic elements. In *Drosophila*, females are more sensitive at lower aluminum concentrations, while the difference in sensitivity between the sexes disappears at higher concentrations [24]. Furthermore, the acute effect of a lower aluminum concentration (120 mg/kg) has a stimulatory effect and increases longevity in *Drosophila*. We emphasize that we followed the chronic treatment from hatching and that it is impossible to separate the larvae by sex at this stage. The responses of the larvae to aluminum treatments T2 and T3 were very different, both in terms of fitness characteristics and locomotion (the lowest concentration of aluminum increased locomotion parameters, which then decreased in T2 and T3, while the lowest concentration reduced mass and RGR, whose values later increased at the same higher metal concentrations noted above). Thus, we can conclude that the larvae are either well adapted to aluminum, owing to the detoxification mechanisms, or that the concentrations used in the experiment were not high enough to induce drastic changes. Since insects can recognize the presence of contaminants, they move more when in search of uncontaminated food [66,67]. It is obvious from our data that larger larvae move less and cover a shorter distance. Avoidance of contaminated food can lead to a decrease in larval mass, changes in relative growth rate, and variability in development time. The average speed while in motion is reduced with an increase in the amount of metal in this organism’s diet. This physiological adaptation probably serves to conserve energy and allows the larvae to cover longer distances. This finding is consistent with the previously observed absence of a characteristic U-shaped dose response and indicates specificity that may be due to differences in the degree of adaptation or the adaptation potential of the locomotion parameters analyzed [68]. Therefore, travel distance and time in movement are prioritized over average speed while in motion during adaptation to aluminum-induced stress. At the highest aluminum concentration, there is a negative correlation between larval mass and all locomotion parameters, indicating that the smallest larvae can travel farther and faster.

The toxicity of aluminum is also due to the imbalance between generated and removed reactive oxygen species (ROS), leading to damage to lipid membranes and proteins, increased ROS synthesis in the mitochondria, and thus damage to mtDNA, along with interference with Ca^2+^ homeostasis in the mitochondria, triggering apoptosis [69]. The toxicity of aluminum also results from the replacement of Mg^+2^ and Fe^+3^ ions, leading to numerous disorders in the body. The replacement of Mg, which is an integral part of the complex with ATP, can impair the activity of the synthesis of enzymes that utilize ATP [70]. All the listed toxic effects of aluminum lead to changes in fitness traits and locomotion. The consequence of these changes is reduced production of the energy required for locomotion and growth.

We must bear in mind that the degree of absorption of this metal is influenced by the age of the organism, the type of compound containing aluminum, the parts of the digestive system, and the medium through which the metal is introduced into the organism [71,72]. In addition, insects have a specific life cycle in which a large proportion of this metal is excreted during molting [44]. We have found significant effects on fitness traits, AChE activity, and locomotion, but further biochemical and molecular biomarkers, relevant detoxification pathways, and the possible adaptations of this species need to be investigated.

## Figures and Tables

**Table 1:** Correlations (Spearman’s correlation coefficient) between the fitness and locomotion traits as well as AChE activity of *L. dispar* larvae exposed to aluminum at concentrations of 50, 250, 500, and 1000 μg Al/g dry food (T1, T2, T3, and T4, respectively) and the control group (C). The intensity of the colors indicates the strength of the correlations. Red and blue indicate negative and positive correlations, respectively.

	Larval Mass	Relative Growth Rate	Development Time	AChE	Travel Distance	Time in Movement	Average Speed in Motion	
Larval mass		−0.314	0.213	−0.257	0.348	0.143	−0.314	C
		−0.371	0.441	0.899	0.257	0.943	−0.257	T1
		0.300	0.564	−0.300	−0.300	−0.300	0.100	T2
		0.700	0.632	0.800	−0.500	0.100	0.300	T3
		0.400	−0.316	0.600	−1.000	−1.000	−1.000	T4
Relative growth rate	−0.314		−0.030	−0.029	0.754	0.086	0.029	C
	−0.371		0.177	−0.406	0.257	−0.314	0.657	T1
	0.300		−0.205	0.800	−0.700	−0.700	−0.600	T2
	0.700		−0.105	0.800	−0.800	−0.400	−0.300	T3
	0.400		−0.632	0.400	−0.400	−0.400	−0.400	T4
Development time	0.213	−0.030		−0.334	0.185	−0.091	0.091	C
	0.441	0.177		0.448	0.088	0.530	0.177	T1
	0.564	−0.205		−0.616	−0.359	−0.359	−0.205	T2
	0.632	−0.105		0.264	0.105	0.527	0.738	T3
	−0.316	−0.632		0.316	0.316	0.316	0.316	T4
AChE	−0.257	−0.029	−0.334		−0.029	0.257	0.029	C
	0.899	−0.406	0.448		0.290	0.986	−0.261	T1
	−0.300	0.800	−0.616		−0.500	−0.500	−0.600	T2
	0.800	0.800	0.264		−0.700	−0.500	−0.300	T3
	0.600	0.400	0.316		−0.600	−0.600	−0.600	T4
Travel distance	0.348	0.754	0.185	−0.029		0.290	−0.232	C
	0.257	0.257	0.088	0.290		0.314	0.771	T1
	−0.300	−0.700	−0.359	−0.500		1.000	0.900	T2
	−0.500	−0.800	0.105	−0.700		0.600	0.300	T3
	−1.000	−0.400	0.316	−0.600		1.000	1.000	T4
Time movement	0.143	0.086	−0.091	0.257	0.290		−0.943	C
	0.943	−0.314	0.530	0.986	0.314		−0.200	T1
	−0.300	−0.700	−0.359	−0.500	1.000		0.900	T2
	0.100	−0.400	0.527	−0.500	0.600		0.900	T3
	−1.000	−0.400	0.316	−0.600	1.000		1.000	T4
Average speed in motion	−0.314	0.029	0.091	0.029	−0.232	−0.943		C
	−0.257	0.657	0.177	−0.261	0.771	−0.200		T1
	0.100	−0.600	−0.205	−0.600	0.900	0.900		T2
	0.300	−0.300	0.738	−0.300	0.300	0.900		T3
	−1.000	−0.400	0.316	−0.600	1.000	1.000		T4

## Data Availability

The raw data supporting the conclusions of this article will be made available by the authors on request.
